# Non-invasive transcranial alternating current stimulation of spatially resolved phosphenes

**DOI:** 10.3389/fnins.2023.1228326

**Published:** 2023-08-17

**Authors:** Faraz Sadrzadeh-Afsharazar, Alexandre Douplik

**Affiliations:** ^1^Photonics Group, Department of Physics, Faculty of Science, Toronto Metropolitan University (Formerly Ryerson University), Toronto, ON, Canada; ^2^Keenan Research Centre of the Li Ka Shing (LKS) Knowledge Institute, St. Michael Hospital, Toronto, ON, Canada

**Keywords:** phosphenes, electrical stimulation, visual perception, transcranial alternating current stimulation (tACS), spatially resolved phosphenes

## Abstract

This study focused on the use of Non-Invasive Transcranial Alternating Current Stimulation (NITACS) to induce and map phosphenes (spark-like percepts in the visual field) in healthy individuals. The study found optimal stimulation parameters to induce reliable phosphenes without skin irritation or pain. The results suggest NITACS can be used as a tool to investigate the relationship between facial stimulation location and phosphene localization within the field of vision (FOV) and raise questions about the origin of phosphenes generated through NITACS. The outcomes of this study could serve as a source of inspiration for creating non-invasive visual aids in the future.

## Introduction

NITACS (Non-Invasive Transcranial Alternating Current Stimulation) is a tool used to study the relationship between cutaneous electrical stimulation of certain facial regions and the experience of phosphenes (visual perceptions) (Oster, 1970; Carvalho et al., 2018; Indahlastari et al., 2018; Liu et al., 2018).

When the facial skin gets subject to an external changing electric field, the receiver of the shock will experience perceptible visual phenomena called phosphenes.

In this study, the focus is on localizing phosphenes within the field of vision (FOV) using NITACS by placing and activating electrodes at different positions around the eye orbits. The study aims to map the location and shape of phosphenes across a healthy population, serving as foundational research for future models (Paulus, 2010; Schutter and Hortensius et al., 2010; Schutter, 2016; Granley and Beyeler et al., 2021).

## Methods

**Figure 1:**
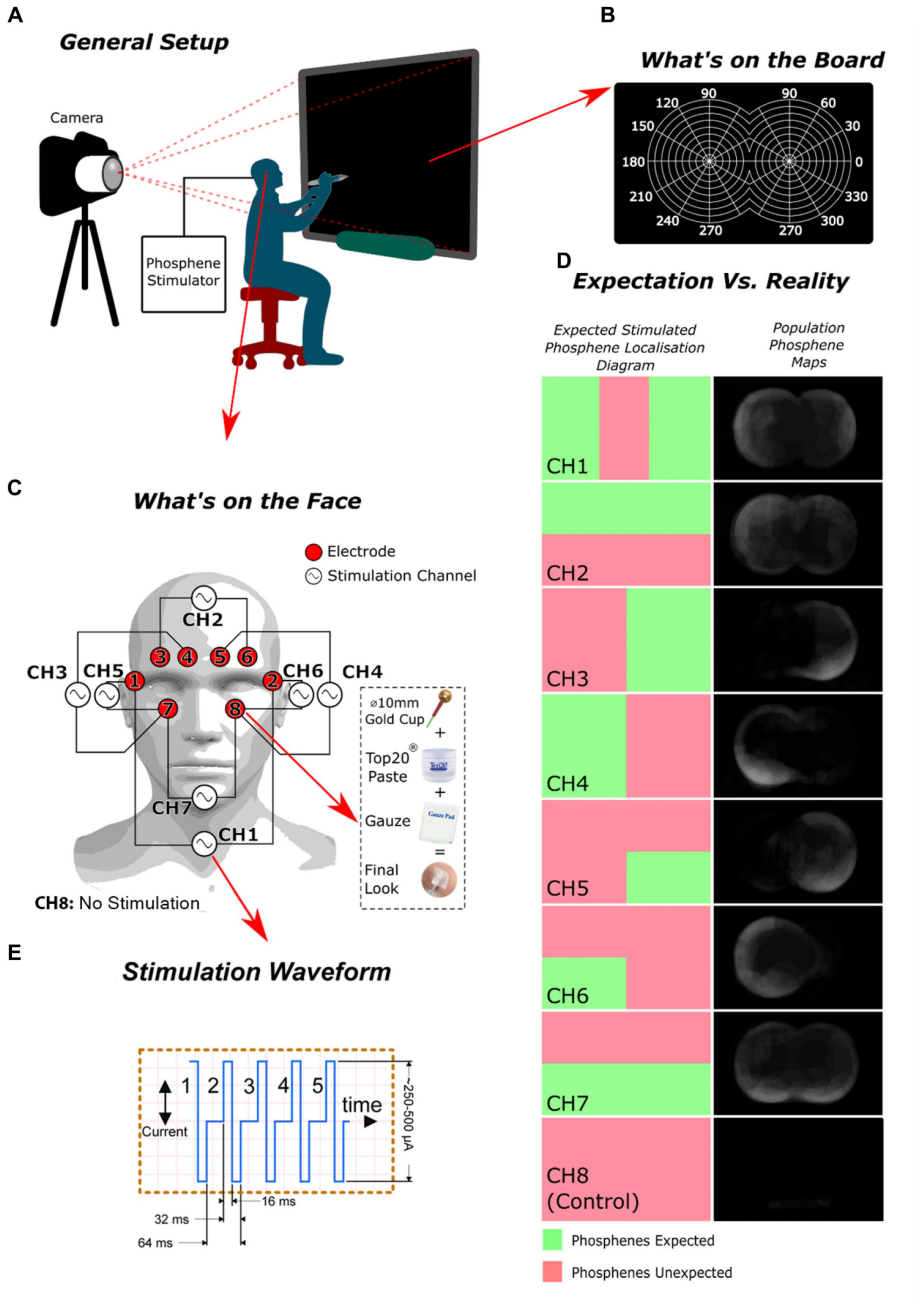
The experiment’s design and outcomes: **(A)** the overall arrangement of the setup; **(B)** the configuration of the smart board employed for participants to indicate the location of the perceived phosphenes they experienced; **(C)** the electrode and stimulation channel configuration used; **(D)** a comparison of the expected spatial distribution of phosphenes versus the observed distribution for each channel; **(E)** The stimulation waveform, comprised of five iterations of a charge-balanced current pulse.

The study (REB: 2019–324) involved eight healthy consenting volunteers (75% males (*n* = 6) | 25% females (*n* = 2) | mean ± SD age: 31.3 ± 14.8) who were each outfitted with an EEG electrode dressing strategically placed around the eye sockets (Figure 1C). The electrodes were hooked up to an in-house custom stimulator (Supplementary Figure S1). The study was conducted in a dimly lit room with each participant with their eyes open sat 50 cm away from a visual perimetry target (Figures 1A–C). The first step involved determining the phosphene stimulation threshold for each participant. The threshold was determined by gradually increasing the stimulation current (from 100 to to 500 μA, of a burst of five 64 ms charge-neutral pulses (Figure 1E)) and asking the participant to report perceiving a phosphene. Once the threshold was found, the stimulation intensity was kept constant for the remainder of the experiment. To avoid pain, the current was precautionarily kept under 500 μA, based on findings from initial studies.

The study collected phosphene drawings from participants and analyzed the results. The drawings were converted into individual phosphene maps for each participant and then into population phosphene maps by averaging the maps from each participant for a specific stimulation channel (complete process depicted in Supplementary Figure S3). Receiver Operating Characteristic (ROC) analysis was applied (complete process depicted in Supplementary Figure S4, Eq S1, and Eq S2) to these maps using the regions where phosphenes are theoretically expected as ground truth (Figure 1D). The ground truths were derived from crude educated guesses, premised on the notion that phosphenes are likely to manifest near the stimulation electrodes.

## Results

In nearly 98% of the non-control stimulation trials, participants consistently described perceiving phosphenes as vibrant rapidly flickering blobs, appearing either as white or occasionally bluish light within their field of vision. Furthermore, every participant confirmed that this was their initial encounter with this specific type of phosphenes, distinct from mechanically induced phosphenes, such as those produced by rubbing the eyes over closed eyelids.

The potential for phosphene maps to remain true to their predicted patterns (concentrated close to the active electrodes) was found to vary among the tested population. The control trial (no stimulation) was 98.4% effective in producing no phosphenes occurrence (complete data in Supplementary Figure S2). While the reasons for the stimulation configuration and electrode placements not producing 100% true positives are unknown, our results suggest that a person’s natural ability to perceive phosphenes may play a role.

Regarding the analysis of channels based on ROC, channels 3 and 4 obtained the most favorable scores, with channels 5 and 7 following behind. The sensitivity/specificity scores were highest for channels 3 and 4 (84.4%|75 and 100%|81.3%, respectively). In contrast, channel 6 had a low sensitivity/specificity score (50%|45.3%), likely due to an unresolved technical problem that compromised consistency across all participants. The results of the control trial showed the highest ROC score, indicating that our stimulation method was effective in rejecting placebo effects. The best-performing phosphene maps (channels 3, 4, 5, 7) generated phosphenes near the stimulation electrodes.

The spatial discrimination of phosphenes was demonstrated for all channels and individuals, with the exception of channel 6. The phosphene maps showed a general trend in appearing in the peripheral visual field, while the central vision showed a general absence of phosphenes.

The hypothesis of spatially encoded phosphenes through electrical stimulation of the face skin assumes that the proximal visual sensing structures will be more stimulated than those of the distal tissues due to higher field strength. To the best of our knowledge, this study presents the first successful attempt at creating spatially resolved phosphenes through facial cutaneous electrical stimulation without surgical intervention or placing electrodes on the eye surface.

### Limitations

The study’s limited sample size constrained its findings; therefore, a more extensive human study will provide greater understanding of the association between cutaneous stimulation sites and spatial characteristics of phosphenes in the visual field.

The study proposed random selection of gender and sex in participant recruitment to provide equity, diversity, and inclusion under condition of the limited number of participants. Future research should address this limitation by including diverse gender identities and sex groups in recruitment strategies. Additional factors, such as age, and ethnicity, should also be considered in future studies to ensure a more comprehensive understanding of the phenomenon.

### Future study

The article investigates the use of non-visual stimuli for creating sensorics responses and the potential benefits of studying individual differences in phosphene perception. The next development is a phosphene stimulator with a camera and machine vision complex (Beck, 1987). Future work aims to induce phosphenes in the central visual field using non-invasive stimulation methods.

## Data availability statement

The raw data supporting the conclusions of this article will be made available by the authors, without undue reservation.

## Ethics statement

The studies involving humans were approved by TMU ethics committee/institutional review board. The studies were conducted in accordance with the local legislation and institutional requirements. The participants provided their written informed consent to participate in this study.

## Author contributions

FS-A designed the stimulator, conducted the human study, analyzed the data, and wrote the manuscript. AD originated the research idea, provided funding, supervised the project, advised on all aspects of the research, edited the manuscript, and prepared it for final submission. All authors contributed to the article and approved the submitted version.

## Conflict of interest

The authors declare that the research was conducted in the absence of any commercial or financial relationships that could be construed as a potential conflict of interest.

## Publisher’s note

All claims expressed in this article are solely those of the authors and do not necessarily represent those of their affiliated organizations, or those of the publisher, the editors and the reviewers. Any product that may be evaluated in this article, or claim that may be made by its manufacturer, is not guaranteed or endorsed by the publisher.
