# Supplementary material for: Non-invasive transcranial alternating current stimulation of spatially resolved phosphenes
Source: Front Neurosci. 2023 Aug 17;17:1228326. doi: 10.3389/fnins.2023.1228326 (PMC10469618; doi:10.3389/fnins.2023.1228326)
Supplement: Supplementary file 1 [file Data_Sheet_1.pdf]

Non-Invasive Transcranial Alternating Current Stimulation (NITACS) of Spatially Resolved Phosphenes – Supplementary Material

Faraz Sadrzadeh-Afsharazar[1] and Alexandre Douplik[1,2*]
[1]Photonics Group, Department of Physics, Faculty of Science, Toronto Metropolitan University (formerly Ryerson University), Toronto, ON, Canada
[2]Keenan Research Centre of the Li Ka Shing (LKS) Knowledge Institute, St. Michael Hospital, Toronto, ON, Canada
*Correspondence to: douplik@torontomu.ca

Keywords: Phosphenes | Electrical Stimulation | Visual Perception

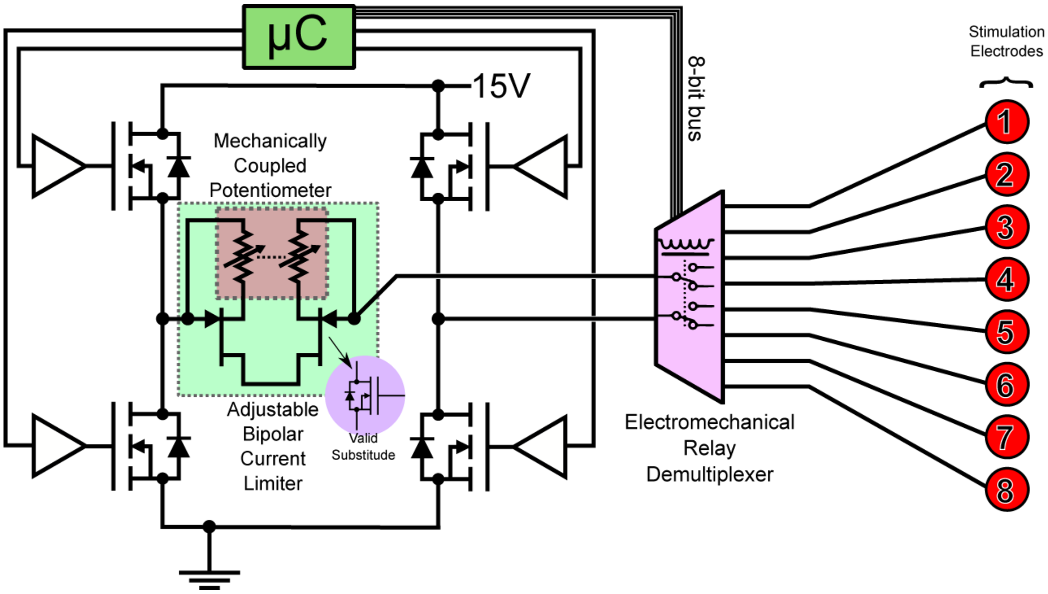

Figure S1. Circuit architecture of the in-house stimulator. Simplified circuit diagram of the 8-electrode phosphene stimulator used in the human study. A monolithic H-bridge (2EDN8524FXTMA1, Infineon) was employed to generate the charge-balanced waveform. A common-sourced depletion-mode field effect transistor pair (LND250, Microchip Inc.) and a dual-ganged 10 k Ω linear potentiometer were used to set the current limit. A set of eight double-pole double-throw signal relays were used to demultiplex the stimulation waveform across eight electrodes.

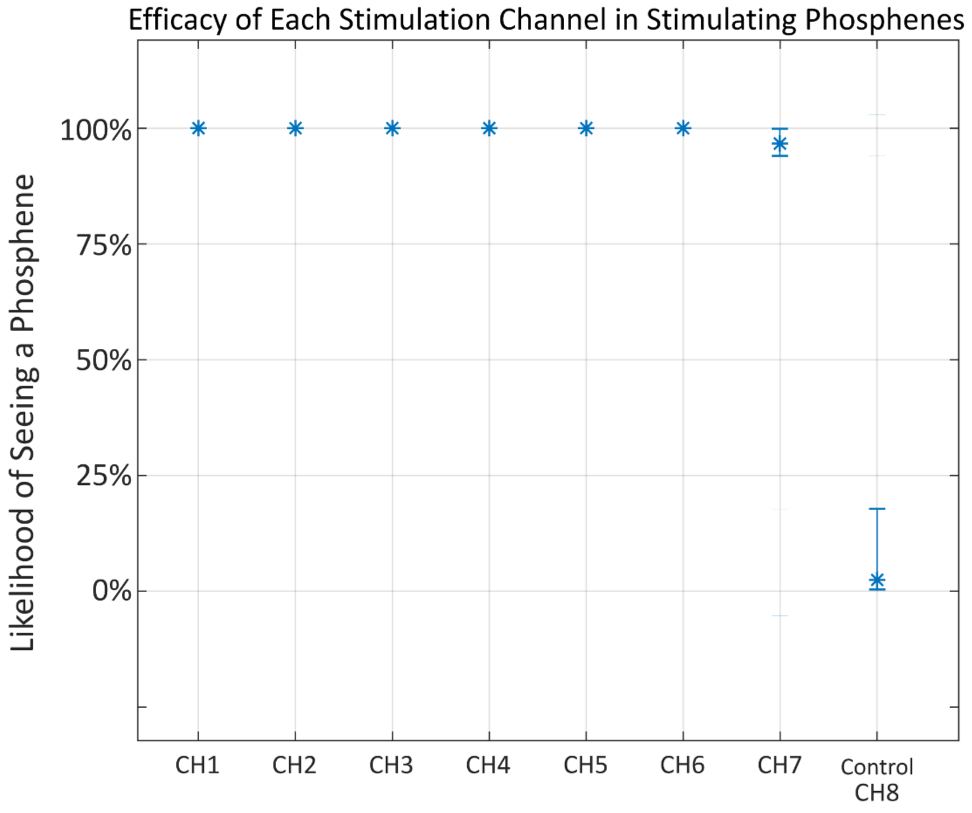

Figure S2. The probability of observing a phosphene during stimulation for each channel across the population is represented as an average value with an error bar range from minimum to maximum. This likelihood is expressed as a percentage of trials where participants produced a drawing without reporting a lack of phosphene perception.

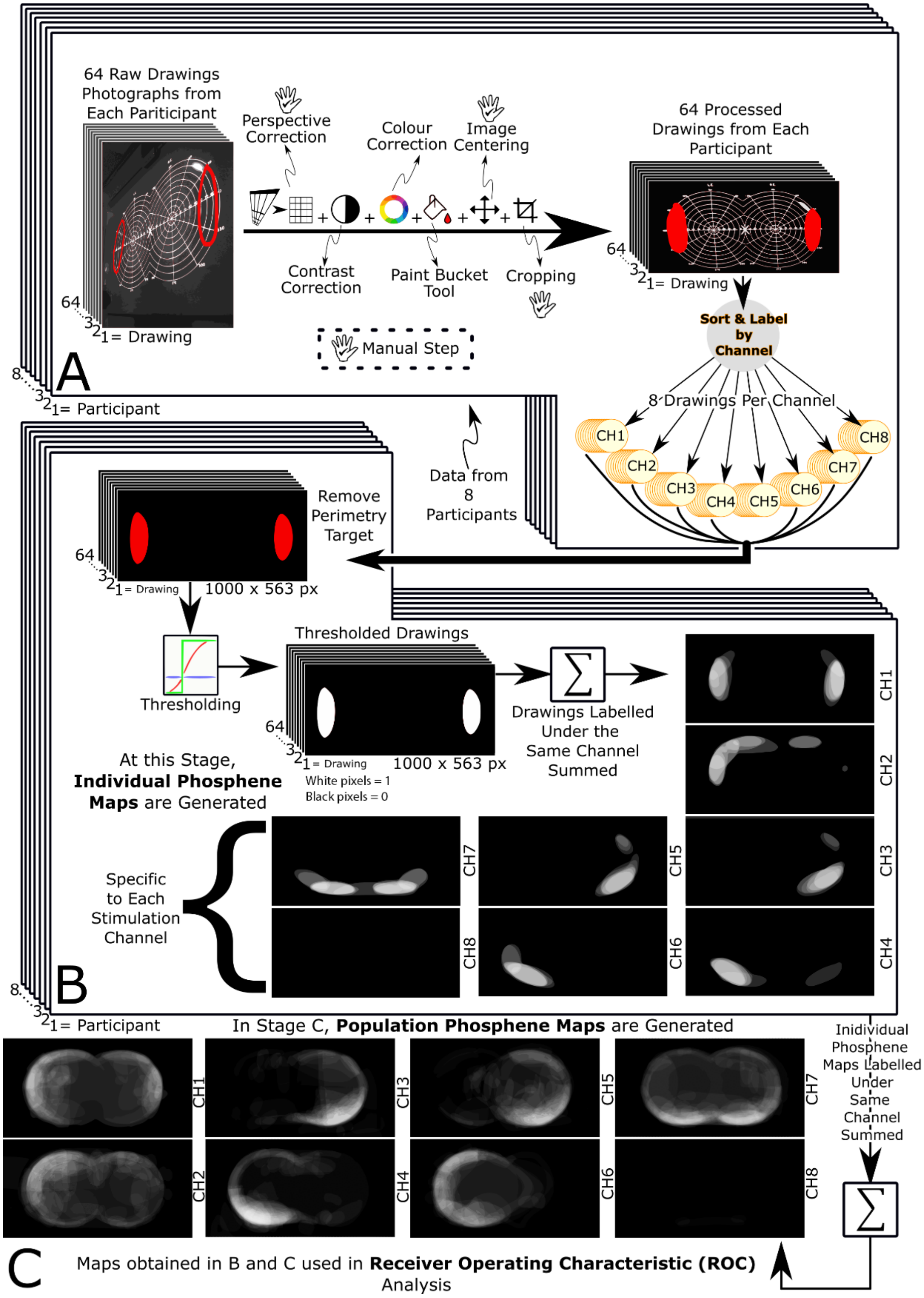

Figure S3. Image processing steps for generating the individual and population phosphene maps from raw drawings: A - Drawings are perspective corrected and drawing contours are filled in; B - Drawn areas are binarized and summed to generate individual phosphene maps; C - Channel specific individual phosphene maps were summed, yielding the population phosphene maps.

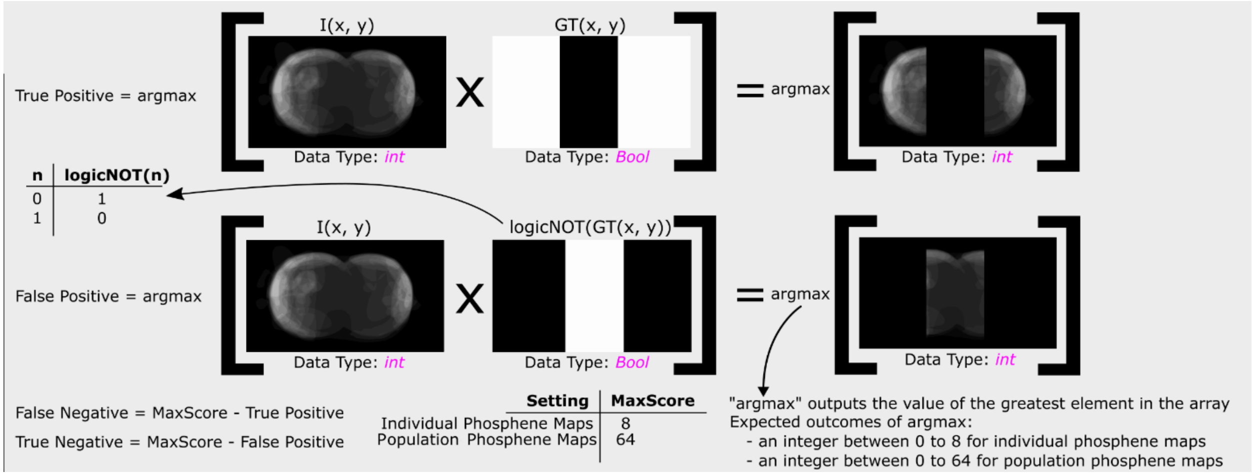

Figure S4. ROC analysis involves computing numerical values that are essential for determining sensitivity and specificity. These values include true positive, true negative, false positive, and false negative.

$$Sensitivity = \left(\frac{True\ Positive}{True\ Positive + False\ Negative}\right) \times 100\% \qquad Eq.S1$$

$$Specificity = \left(\frac{True\ Negative}{True\ Negative + False\ Positive}\right) \times 100\% \qquad Eq.S2$$
